# DNA Methylation and Fetal Programming of Cardiovascular Disease: From Congenital Heart Diseases to Adult Cardiovascular Dysfunction

**DOI:** 10.31083/RCM46174

**Published:** 2026-04-02

**Authors:** Zewen Chen, Min Qiu, Yifan Li, Wen Xie, Tianyu Chen, Hailong Qiu, Yong Zhang, Shusheng Wen

**Affiliations:** ^1^Department of Cardiac Surgery, Guangdong Cardiovascular Institute, Guangdong Provincial People's Hospital (Guangdong Academy of Medical Sciences), Southern Medical University, 510080 Guangzhou, Guangdong, China; ^2^Department of Pediatric Cardiology, Guangdong Cardiovascular Institute, Guangdong Provincial People's Hospital (Guangdong Academy of Medical Sciences), Southern Medical University, 510080 Guangzhou, Guangdong, China; ^3^Department of Cardiology, Inselspital, Bern University Hospital, University of Bern, 3010-CH Bern, Switzerland

**Keywords:** DNA methylation, fetal programming, congenital heart disease, cardiovascular disease

## Abstract

DNA methylation, the most extensively studied epigenetic mechanism, acts as a critical interface between maternal environmental influences and fetal cardiovascular development. During embryogenesis, tightly orchestrated methylation remodeling regulates the transcriptional networks required for cardiogenesis, including chamber septation, valve formation, and myocardial maturation. Disruption of these methylation patterns contributes to congenital heart disease (CHD), with distinct methylation signatures identified in tetralogy of Fallot, double-outlet right ventricle, bicuspid aortic valve, and coarctation of the aorta. Maternal exposures, including smoking, alcohol intake, folic acid status, hypertension, diabetes, and hyperlipidemia, modify fetal DNA methylation in placental, myocardial, and cord blood tissues. These alterations affect key developmental pathways, including Wnt, Notch, and mitogen-activated protein kinase signaling, as well as genes that regulate oxidative stress, thereby increasing the risk of structural defects and predisposing offspring to long-term cardiovascular vulnerability. Epigenetic reprogramming in adverse intrauterine environments has been linked to hypertension, pulmonary vascular disease, atherosclerosis, and ischemia-sensitive phenotypes in later life, supporting a continuum from fetal life to adult cardiovascular dysfunction. Unlike genetic mutations, DNA methylation is dynamic and reversible, highlighting the potential of this modification as a biomarker of early risk and a target for preventive strategies. Optimizing maternal health, ensuring appropriate folate intake, and reducing harmful exposures may help preserve normal methylation landscapes and improve offspring cardiovascular outcomes. Advances in high-resolution epigenomic profiling, including single-cell methylation technologies, now enable delineation of cell-specific trajectories that connect CHD with adult cardiovascular disease and may inform precision interventions aimed at modifying pathogenic epigenetic states. Thus, understanding the role of DNA methylation in fetal programming can clarify the developmental origins of CHD and adult cardiovascular disorders, and lay the foundation for cardiovascular prevention strategies that extend from preconception through the earliest stages of life.

## 1. Introduction

Epigenetics, a concept first articulated by Conrad Waddington in 1939 from the Greek term “epigenesis”, was originally intended to describe the broad connection between genetics and developmental biology, closely paralleling the principles of embryology [1]. Over subsequent decades, the definition of epigenetics has become more precise, now encompassing heritable structural and biochemical modifications of chromatin that regulate gene expression without altering the underlying nucleotide sequence [2]. Epigenetic regulation occurs through multiple mechanisms, including DNA methylation, histone modification, and the activity of non-coding RNAs, which collectively orchestrate transcriptional control and chromatin accessibility [3]. These mechanisms establish and maintain cellular identity, allowing genetically identical cells to acquire distinct functional phenotypes during differentiation and throughout cell division [4]. Dysregulation of these processes has been increasingly recognized as a contributor to a wide range of diseases, particularly cardiovascular disease (CVD) [5].

Among the spectrum of CVD, congenital heart disease (CHD) represents the most prevalent congenital malformation worldwide [6, 7]. Current estimates suggest a prevalence of 8–10 cases per 1000 live births [7, 8, 9], a figure that continues to rise with improvements in diagnostic accuracy, particularly through advances in echocardiography and fetal imaging [8, 10, 11]. The etiology of CHD is multifactorial, reflecting the complex interplay of chromosomal abnormalities, gene variants, maternal metabolic disorders such as gestational diabetes mellitus (GDM) and obesity, pregnancy-induced hypertension, and environmental exposures including tobacco smoke and drug intake [12]. Collectively, these risk factors lead to structural intracardiac anomalies or comorbid conditions that account for up to 30% of fetal deaths [13]. The substantial morbidity and mortality associated with CHD highlight the urgent need to identify mechanisms beyond classical genetics that govern gene regulation and mediate the influence of maternal and environmental factors on cardiac development.

Epigenetic regulation, particularly DNA methylation, offers a compelling framework for understanding how environmental cues are integrated into developmental gene expression programs. Unlike permanent genetic mutations, epigenetic modifications are dynamic, potentially reversible, and responsive to external stimuli, rendering them both mechanistic insights and therapeutic opportunities. Growing evidence has demonstrated that alterations in DNA methylation play a central role in cardiac development, CHD pathogenesis, and the broader spectrum of cardiovascular dysfunction [14].

In this review, we examine the role of DNA methylation in cardiovascular development and diseases. Specifically, we focus on four aspects: the molecular mechanisms of DNA methylation during cardiogenesis; the influence of maternal factors, including smoking, alcohol exposure, and folic acid intake, on fetal cardiac epigenetic programming; the associations between aberrant DNA methylation and specific CHD subtypes; and the implications of fetal epigenetic programming for cardiovascular dysfunction in later life. By synthesizing mechanistic, epidemiologic, and translational evidence, we aim to provide new perspectives on prevention, early intervention, and potential therapeutic strategies for cardiovascular disease across the lifespan.

## 2. DNA Methylation in Cardiac Development

DNA methylation was first reported in 1925 by Johnson and Coghill in bacteria [15]. Although the modification was recognized early, its biological importance remained unclear for decades. Only with the accumulation of extensive research through the twentieth century did its critical role in mammalian development become evident, particularly from the 1990s onward [16]. Cardiomyocytes arise from progenitor cells during early embryogenesis and subsequently mature with a limited capacity for regeneration. As a result, cardiomyocytes rely on tightly regulated developmental programs to adapt to contractile and metabolic demands. These programs are characterized by specific patterns of gene expression that are established during development and sustained through fetal programming [17]. DNA methylation has emerged as a central regulator of this process, modulating cardiac gene expression and influencing both normal development and susceptibility to disease [18].

DNA methylation occurs predominantly at CpG dinucleotides, where a cytosine is followed by a guanine, and these sites are frequently methylated. While most genomic regions are CpG-poor, CpG islands represent clusters enriched in CpG sites that are generally unmethylated. Notably, nearly 70 percent of mammalian promoters are embedded within CpG islands, permitting transcription factor binding and transcriptional activation. This distribution highlights the fundamental role of methylation patterns in regulating gene expression [14, 19]. The remodeling of DNA methylation in a precise temporal and spatial manner is integral for embryogenesis, including cardiogenesis, across different developmental stages [20]. CpG methylation therefore contributes not only to the regulation of gene expression but also to developmental programming and genomic stability [21].

These modifications are catalyzed by a coordinated enzymatic system. Maintenance methylation is mediated primarily by DNA methyltransferase 1 (DNMT1), while de novo methylation is carried out by the DNMT3 family [22]. Active demethylation involves the oxidation of 5-methylcytosine by the ten-eleven translocation (TET) family of enzymes (TET1, TET2, and TET3) [23]. DNMT3A and DNMT3B have been implicated as key contributors to the pathogenesis of CHD [24], whereas DNMT1 plays an essential role in maintaining gene expression programs in embryonic cardiomyocytes [25].

The function of the TET family in cardiac development is less well understood, yet accumulating evidence indicates their importance. In murine models, embryos deficient in *TET1*, *TET2*, and *TET3* at embryonic days 8.0 to 8.5 exhibited hyperactivation of the Wnt pathway, which diverted bipotent neuromesodermal progenitors toward mesodermal lineages at the expense of neuroectoderm formation [26]. Deletion of *TET1*, *TET2*, and *TET3* also resulted in a failure of cardiomyocyte generation from human embryonic stem cells (ESCs) [27]. Within the cardiovascular system, cardiac-specific deletion of *TET2* and *TET3* produced ventricular non-compaction cardiomyopathy and embryonic lethality, driven by impaired DNA demethylation and reduced chromatin accessibility [28]. In zebrafish, *TET2* and *TET3* activity has been shown to be critical for cardiac development, particularly through recruitment of epicardial progenitors during atrioventricular canal formation [29]. Consistent with these findings, deficiency of TET proteins in human and murine embryonic stem cells leads to promoter hypermethylation and dysregulation of essential developmental genes [30, 31].

Between embryonic days 11.5 and 14.5, cardiac cells undergo extensive differentiation, migration, and proliferation, processes required for morphogenesis of the cardiac chambers, septation, valve formation, myocardial compaction, and coronary vasculature [32, 33]. These events are orchestrated by transcriptional networks and endocardial–myocardial signaling pathways [34]. Although global methylation remains relatively stable across 1.64 million CpG sites in mouse hearts during this window, 2901 sites exhibit dynamic methylation changes. Enrichment analyses demonstrate that these sites map to genes involved in critical aspects of heart development, including Erb-B2 receptor tyrosine kinase 4 (*ERBB4*), GATA binding protein 6 (*GATA6*), forkhead box P1 (*FOXP1*), fibroblast growth factor 9 (*FGF9*), myocyte enhancer factor 2C (*MEF2C*), roundabout guidance receptor 2 (*ROBO2*), Wnt family member 2 (*Wnt2*), and hyaluronan synthase 2 (*HAS2*) are included [35].

Each of these genes illustrates the intersection of methylation and cardiac morphogenesis. *ERBB4* regulates ventricular myocardial growth during prenatal development and contributes to postnatal cardiomyocyte proliferation, myocardial homeostasis, and even prognosis of heart failure in human populations [11, 36]. *GATA6* is a master regulator of cardiac morphology, with abnormal expression leading to atrioventricular canal malformations and outflow tract defects [37]. It is also essential for pacemaker cell differentiation and conduction system development [38]. *FOXP1* governs cardiomyocyte proliferation and heart development and has been shown to support post-injury regeneration [39]. Within the fibroblast growth factor family, *FGF9* is required for cardiomyocyte proliferation, whereas *MEF2C* serves as a core transcription factor driving cardiomyocyte differentiation and activation of the cardiac program [40]. *ROBO2*, though less extensively studied, participates in cardiac cell migration, chamber and septal morphogenesis, and valve development, and its dysfunction has been linked to bicuspid aortic valve (BAV) [41, 42]. *Wnt2* signaling regulates cell fate decisions within the second heart field, balancing proliferation and differentiation during early cardiogenesis [43].

**Fig. 1.**
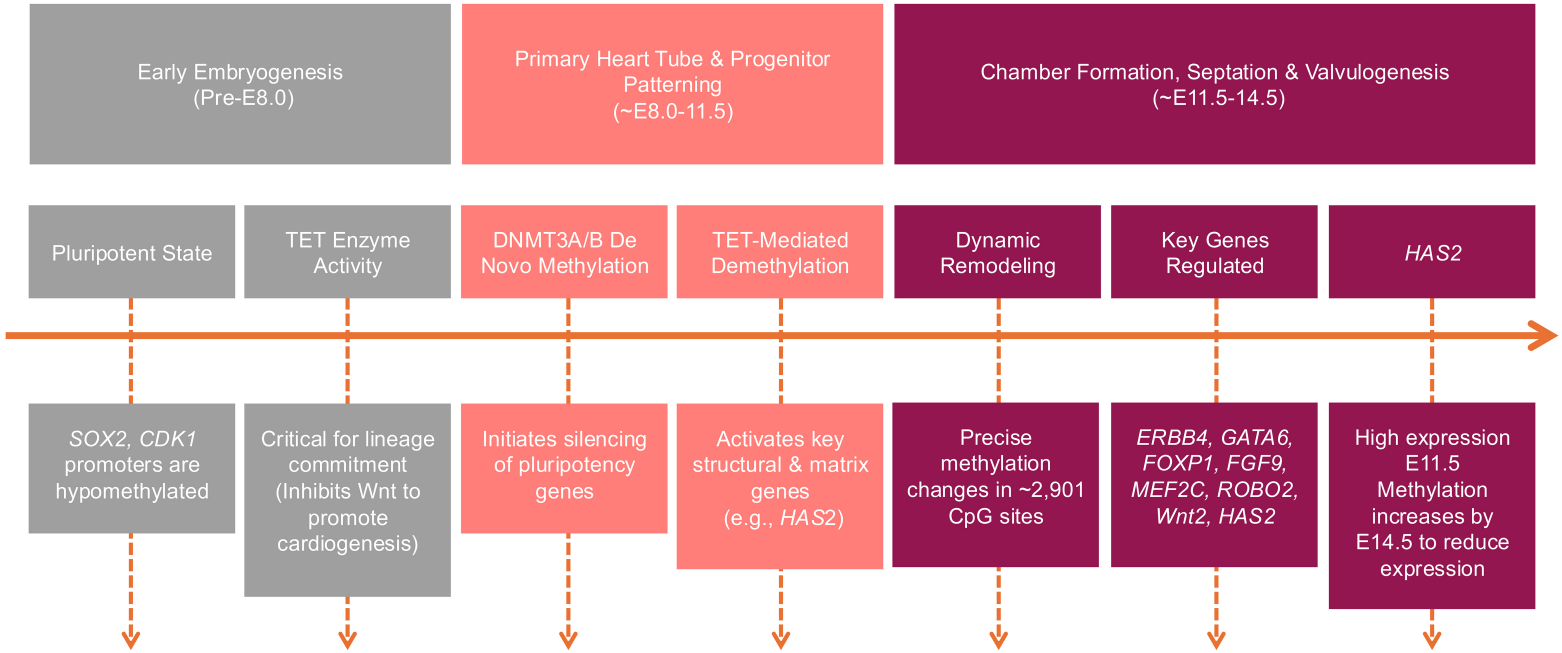
**DNA methylation in cardiogenesis**. TET, ten-eleven translocation; DNMT, DNA methyltransferase; *HAS2*, hyaluronan synthase 2; *SOX2*, SRY-Box transcription factor 2; *CDK1*, cyclin dependent kinase 1; *ERBB4*, Erb-B2 receptor tyrosine kinase 4; *GATA6*, GATA binding protein 6; *FOXP1*, forkhead box P1; *FGF9*, fibroblast growth factor 9; *MEF2C*, myocyte enhancer factor 2C; *ROBO2*, roundabout guidance receptor 2; *Wnt2*, wnt family member 2.

*HAS2* provides a further example of epigenetic regulation in cardiac development. *HAS2* synthesizes hyaluronic acid, a critical extracellular matrix component of the cardiac jelly [44]. It is essential for endocardial cushion formation, valve development, and septation, particularly around embryonic day 11.5 [35, 45, 46]. Functional network analyses have identified *HAS2* as interacting with other key regulators, including T-box transcription factor 20 (*TBX20*), essential for endocardial cushion remodeling [47], Heart and neural crest derivatives expressed 1 (*HAND1*), critical for ventricular morphogenesis [48], and Gap junction protein gamma 1 (*GJC1*), which encodes a gap junction protein important for morphogenesis and conduction [49]. Loss of *HAS2* results in embryonic lethality due to absence of endocardial cushion and valve formation, a defect not compensated for by the related enzymes *HAS1* and *HAS*3 [50]. Notably, *HAS2* expression is significantly higher at embryonic day 11.5 than at day 14.5, in parallel with increased methylation at enhancer regions later in development [35]. Inhibition of DNA methylation through DNMT3B repression induces upregulation of *HAS2* expression in developing heart valves at both embryonic days 11.5 and 14.5 [35], suggesting that DNA methylation serves as a key regulator of *HAS2* activity during valve and septal development (Fig. 1).

## 3. Maternal Effects on Cardiac DNA Methylation

DNA methylation is a central mechanism regulating cardiac development through the control of cardiac-specific genes. This raises an important question: what influences the establishment of these methylation patterns during embryogenesis? The maternal environment represents the first and most critical exposure for the developing embryo. Numerous studies have linked abnormal cardiac and great vessel structures to maternal conditions, suggesting that the maternal milieu can directly shape the epigenetic landscape of the offspring. The interaction between maternal conditions and DNA methylation may therefore serve as a critical determinant of cardiac morphogenesis and disease risk. Given that many current gaps in understanding cardiac development stem from limited knowledge about modifiable maternal factors, advancing our insights in this area is essential for designing primary prevention strategies for CVD/CHD.

### 3.1 Maternal Smoking During Pregnancy (MSDP)

MSDP has long been recognized as a major risk factor for adverse fetal outcomes. Nicotine and other toxic compounds generated by smoking can readily cross the placenta, enter the fetal circulation, and accumulate in developing tissues, thereby interfering with normal cellular differentiation and growth [51]. Despite global public health campaigns, MSDP remains prevalent in certain populations, with rates between 10.5% and 16% in Western populations [52]. Beyond its well-known associations with low birth weight and perinatal morbidity, MSDP has now been directly linked to alterations in DNA methylation that contribute to both congenital malformations and long-term cardiovascular vulnerability in offspring [53, 54]. Importantly, epidemiologic data have demonstrated that MSDP increases the risk of CHD, highlighting its relevance to cardiac morphogenesis [55].

**Fig. 2.**
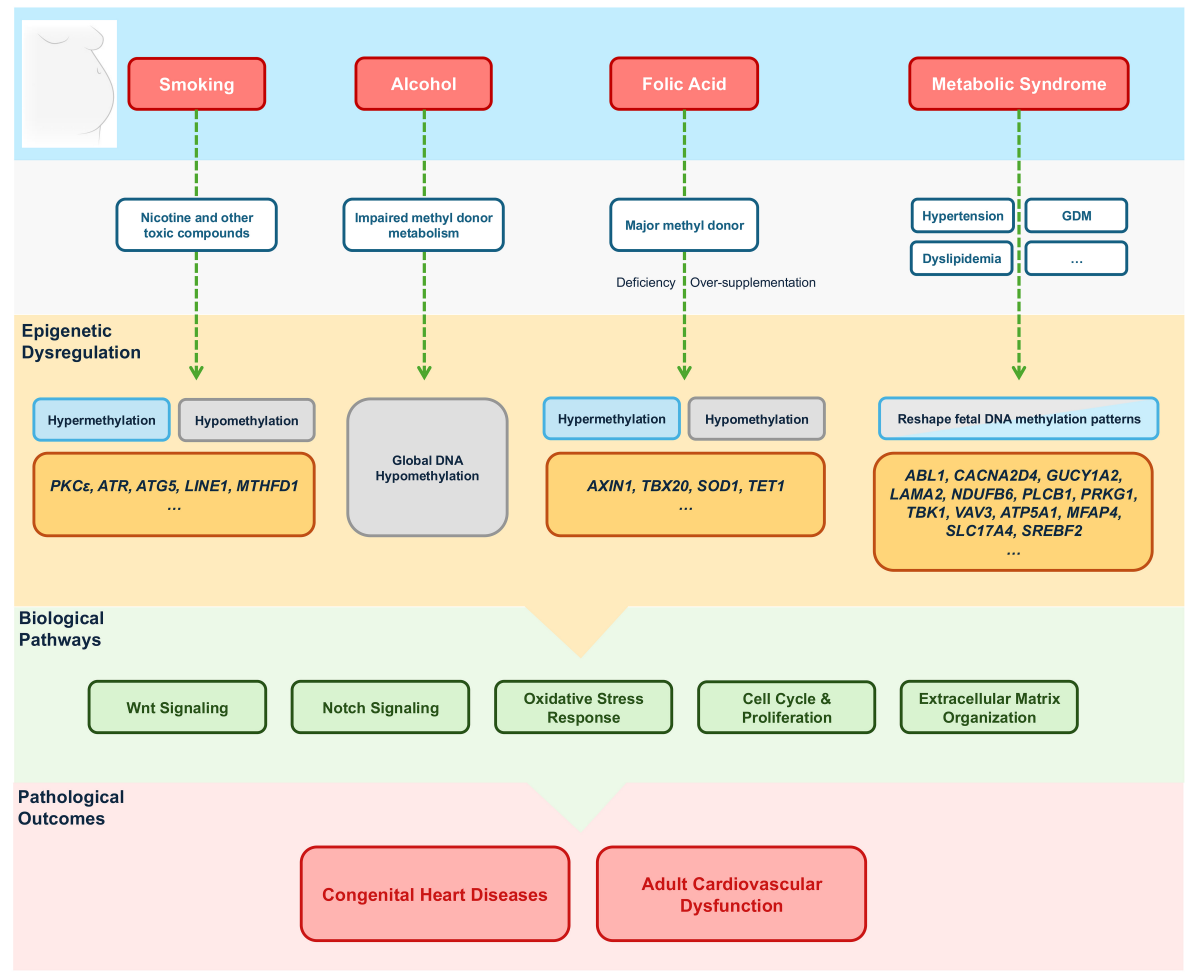
**Maternal effects on cardiac DNA methylation**. GDM, gestational diabetes mellitus; *PKCε*, protein kinase C epsilon; *ATR*, ataxia telangiectasia and rad3-related; *ATG5*, autophagy related 5; *LINE1*, long interspersed nuclear element-1; *MTHFD1*, methylenetetrahydrofolate dehydrogenase 1; *AXIN1*, axis inhibition protein 1; *TBX20*, T-box transcription factor 20; *SOD1*, superoxide dismutase 1; *TET1*, tet methylcytosine dioxygenase 1; *ABL1*, v-abl Abelson murine leukaemia viral oncogene homologue 1; *CACNA2D4*, calcium voltage-gated channel auxiliary subunit alpha2delta 4; *GUCY1A2*, guanylate cyclase 1 soluble subunit alpha 2; *LAMA2*, laminin subunit alpha 2; *NDUFB6*, NADH dehydrogenase (ubiquinone) 1beta subcomplex 6; *PLCB1*, phospholipase C beta 1; *PRKG1*, protein kinase cGMP-dependent 1; *TBK1*, TANK-binding kinase 1; *VAV3*, vav guanine nucleotide exchange factor 3; *ATP5A1*, ATP synthase F1 subunit alpha; MFAP4, microfibril associated protein 4; *SLC17A4*, solute carrier family 17 member 4; *SREBF2*, sterol regulatory element binding transcription factor 2.

Large-scale studies have provided molecular insights into this relationship. In a cohort of 5648 newborns, including 897 with sustained MSDP exposure, investigators identified 5547 CpG sites with significant differential methylation [56]. Among these CpGs, 45% were hypermethylated and 55% hypomethylated compared with unexposed controls [57, 58]. On average, CpGs with higher methylation increased by 0.8%, while CpGs with lower methylation decreased by 0.6% in exposed infants [56]. These seemingly modest changes have broad functional consequences because they occur in genes essential for cardiac homeostasis. For example, MSDP increased promoter methylation of protein kinase C epsilon (*PKCε*) in fetal hearts, leading to heightened susceptibility to ischemia–reperfusion injury [59]. Similarly, MSDP reduced DNA methylation in the promoters of ataxia telangiectasia and rad3-related (*ATR*) and autophagy related 5 (*ATG5*), which correlated with increased offspring susceptibility to ischemia–reperfusion injury and pulmonary hypertension, respectively [60, 61]. Furthermore, MSDP interacts with polymorphisms in the methylenetetrahydrofolate dehydrogenase 1 (*MTHFD1*) gene, a key regulator of one-carbon metabolism, thereby modifying DNA synthesis and methylation and influencing CHD risk [62]. Global methylation markers also support this association: hypomethylation of long interspersed nuclear element-1 (*LINE1*), a surrogate for global DNA methylation, has been linked to increased CHD risk [63], and MSDP has been shown to contribute to this hypomethylated state [64]. Together, these findings suggest that MSDP disrupts both gene-specific and global methylation programs, creating a molecular substrate for congenital and postnatal cardiovascular disease in late life (Fig. 2).

### 3.2 Prenatal Alcohol Exposure (PAE)

PAE also represents a major modifiable maternal risk factor with significant implications for embryonic development. Ethanol freely crosses the placental barrier, and because the fetus lacks the enzymatic machinery for alcohol detoxification, exposure results in prolonged accumulation of ethanol and its metabolites within fetal tissues [65]. A clinical and epidemiological study has established a strong link between PAE and structural anomalies, including CHD [66]. Approximately 28% of infants with fetal alcohol spectrum disorders are affected by CHD, with ventricular septal defect (VSD) being the most common lesion, followed by atrial septal defect (ASD) and tetralogy of Fallot (TOF) [67]. Mechanistic studies suggest that alcohol disrupts cardiac morphogenesis by impairing the function of second heart field progenitors and neural crest cells, both of which play essential roles in endocardial cushion formation, ventricular septation, and outflow tract development [68, 69].

Epigenetic analyses provide further insight into these effects. Like MSDP, PAE has been shown to induce widespread alterations in newborn DNA methylation [70]. The mechanisms are multifaceted, involving both impaired methyl donor metabolism and direct inhibition of DNA methyltransferase activity [71]. In the developing embryo, PAE reduces folate and vitamins B6 and B12, thereby diminishing S-adenosylmethionine, the universal methyl donor for DNA methylation [72]. In both *in vivo* and *in vitro* studies, ethanol has been shown to deplete methyl-donors, leading to global DNA hypomethylation [73]. An additional study has shown that PAE alters methylation of imprinted genes and genes regulating cell cycle, growth, and apoptosis [74]. A more recent study has shown that PAE reduces global DNA methylation in the developing heart at later stages, particularly around incubation day 8, and that this reduction is associated with structural malformations, most prominently hypoplastic right ventricle [69]. Notably, supplementation with glutathione was found to restore the activity of S-adenosylmethionine synthetase, normalize DNA methylation, and reduce the incidence of CHD in this setting [69]. These findings underscore the mechanistic role of DNA methylation in mediating the teratogenic effects of alcohol and suggest potential avenues for therapeutic intervention.

### 3.3 Folic Acid (FA)

FA is a vital nutrient that plays a critical role in nucleotide synthesis, cellular proliferation, and tissue growth. During pregnancy, maternal demand for FA increases substantially to support rapid embryonic and fetal development, highlighting its importance in morphogenesis and organogenesis [75]. As an essential component of the one-carbon metabolism pathway, FA serves as a major methyl donor, directly influencing DNA methylation and gene expression [76]. Genes regulated by FA exhibit distinct methylation changes, either through promoter methylation or through bimodal patterns characterized by low promoter and high gene body methylation [77]. Importantly, FA supplementation has been shown to mitigate the adverse epigenetic effects of PAE, preserving normal DNA methylation and protecting against CHD [69]. Both deficiency and excess of FA have been linked to adverse cardiovascular outcomes in offspring. Maternal FA deficiency has been associated with cardiomyopathy in offspring exposed to a high-fat diet [78], while over-supplementation has been linked to impaired cardiac function through hypermethylation-mediated suppression of *SOD1*, a gene critical for oxidative stress defense [79].

The importance of FA in CHD prevention is supported by a growing body of evidence, including investigations of key genes regulating folate metabolism [80, 81]. Hypomethylation of the axis inhibition protein 1 (*AXIN1*) promoter and hypermethylation of the *TBX20* promoter have been associated with FA deficiency and VSD in offspring [82]. Interestingly, both deficient and excessive maternal FA intake have been associated with hypermethylation of the *TET1* promoter [83]. Given the established role of *TET1* in atrioventricular canal formation [29], these findings underscore the sensitivity of cardiac development to maternal FA status and the central role of DNA methylation in mediating its effects.

## 4. DNA Methylation Patterns and Congenital Heart Disease

CHD encompasses a heterogeneous group of structural cardiac malformations that arise predominantly during prenatal development, a critical period when spatiotemporal regulation of signaling pathways is essential for proper cardiogenesis [84]. DNA methylation plays a pivotal role in this process, influencing the transcriptional regulation of genes involved in heart development and maturation [20]. More importantly, maternal factors that affect fetal DNA methylation patterns are increasingly recognized as key as determinants of offspring cardiac outcomes. Understanding how these epigenetic alterations in DNA methylation contribute to CHD development offers novel insights into disease mechanisms and potential avenues for prevention and intervention.

### 4.1 Common Types of Congenital Heart Disease

CHD is classified into various subtypes based on anatomical and physiological characteristics, ranging from conotruncal anomalies to left ventricular outflow tract (LVOT) anomalies and other malformations like isolated atrial or ventricular septal defects [85]. These classifications provide clinical clarity and offer a framework for investigating the underlying developmental and epigenetic mechanisms contributing to the observed phenotypic variability.

The conotruncus, comprising the conus and the truncus, is a critical embryonic structure. The conus develops into the right ventricular outflow tract (RVOT) and LVOT, while the truncus gives rise to the great arteries [86]. Abnormalities of these regions lead to conotruncal anomalies. TOF is the most frequent conotruncal defect and accounts for approximately 10% of all CHD. TOF is characterized by outflow tract stenosis or atresia. Double-outlet right ventricle (DORV), which represents fewer than 3% of cases, involves abnormal ventriculoarterial alignment. Truncus arteriosus (TA), occurring in 2% to 4% of patients, is defined by outlet septation defects [20, 87, 88].

LVOT anomalies include obstructive malformations of the left heart and aorta, such as hypoplastic left heart syndrome (HLHS), BAV, coarctation of the aorta (CoA), and interrupted of the aortic arch (IAA) [89]. HLHS, characterized by a dominant right ventricle and a hypoplastic left ventricle with diminutive left-sided structures, represents the most severe single-ventricle lesion, although it is rare with a prevalence of less than 0.02% [90]. By contrast, BAV is the most common congenital cardiac malformation, affecting 0.5% to 1.4% of the general population [91]. The morphological spectrum of BAV encompasses a range of valvular malformations, spanning from complete absence of commissures to partial underdevelopment of one or more commissures and asymmetric cusp formation [92, 93]. CoA is the most frequent congenital aortic disorder in children, with an incidence of approximately 3 cases per 10,000 live births. Both CoA and IAA can occur as isolated anomalies or in association with other cardiac malformations, the most common of which are HLHS, aortic stenosis, and VSD [94].

Accumulating evidence suggests that maternal factors can influence DNA methylation during embryogenesis, thereby shaping heart development. Building on this knowledge, investigations into aberrant methylation profiles in CHD represent a promising research direction that may help to unravel the complex mechanisms underlying these conditions.

### 4.2 DNA Methylation Profiling in Congenital Heart Disease

Genome-wide DNA methylation analyses have provided important insights into how maternal epigenetic status may shape fetal cardiac development. The earliest evidence came from a study in 2011 comparing mothers of children with CHD to controls, identifying 425 differentially methylated CpG sites (DMCs), with 90.8% located in CpG islands and nearly 90% hypermethylated in case mothers, while only 10.8 percent were hypomethylated [95]. These findings suggest that maternal DNA methylation patterns may contribute to fetal cardiac development, modifying disease susceptibility.

In affected offspring, large-scale profiling has demonstrated widespread differentially methylated regions (DMRs) across multiple CHD subtypes. Individuals with TOF displayed 14,403 hypermethylated and 1450 hypomethylated DMRs, while those with VSD exhibited 7152 hypermethylated and 935 hypomethylated regions [96]. Additional analyses across pulmonary atresia (PA), TOF, total anomalous pulmonary venous connection (TAPVC), and patent ductus arteriosus (PDA) revealed DMCs enriched in promoters and CpG islands, with hypermethylation predominating [97]. Placental studies similarly identified distinct DMCs and DMRs in CHD pregnancies, suggesting that aberrant methylation signatures are present across multiple fetal-derived tissues [98].

Genetically predisposed populations further illustrate the contribution of epigenetic variation to phenotypic heterogeneity. Approximately half of individuals with Down syndrome develop CHD, and comparative profiling has shown markedly different methylation landscapes between Down syndrome patients with and without cardiac malformations, with 35% hypermethylated and 65% hypomethylated DMRs distinguishing the two groups [99]. These findings suggest that DNA methylation signatures may distinguish phenotypic subgroups even within genetically predisposed populations, reinforcing the role of epigenetic plasticity in the maternal–embryonic–adult disease continuum.

### 4.3 Differentially Methylated Genes Profiling in Congenital Heart Diseases

DMCs and DMRs in CHD have been enriched near genes governing DNA binding and transcription factor activity, consistent with their regulatory potential [100, 101]. In mothers of children with CHD, 425 DMCs mapped to 415 genes. Functional enrichment revealed roles in nucleic acid metabolism, signal transduction, anatomical development, and multicellular organization, underscoring their potential contribution to cardiac morphogenesis [95]. In fetus with CHD, gene biological process enrichment analysis of DMCs pointed to gene networks involved in cardiac development, while pathway analysis of DMCs highlighted aberrant regulation of the Wnt and adrenergic signaling pathways, both of which are critical for normal heart formation [98]. Studies of monozygotic twins provide particularly compelling evidence, as these individuals share nearly identical genomes. In twins with Down syndrome who were discordant for CHD, 197 DMRs were identified in those discordant for VSD and 88 DMRs in those discordant for atrioventricular septal defect (AVSD). Thirteen DMRs were common across both subgroups. Genes such as CBFA2/RUNX1 partner transcriptional co-repressor 3 (*CBFA2T3*), EPH receptor A8 (*EPHA8*), lymphocyte antigen 9 (*LY9*), and solute carrier family 9 member A3 regulator 2 (*SLC9A3R2*) displayed promoter methylation differences that may underlie divergent cardiac phenotypes despite genetic identity [102].

Specific CHD subtypes also exhibit distinct epigenetic signatures (Table 1). In TOF, aberrant methylation was observed in NK2 homeobox 5 (*NKX2-5*) and *HAND1*, with hypermethylation in the gene body and promoter regions, respectively [103]. Correlative analyses linked promoter hypermethylation of epidermal growth factor receptor (*EGFR*), EvC ciliary complex subunit 2 (*EVC2*), T-box transcription factor 5 (*TBX5*) and cripto, FRL-1, cryptic family 1B (*CFC1B*) to altered mRNA expression, implicating these genes in TOF development [104]. Placental studies of TOF cases have identified differential methylation in pathways such as nuclear factor-κB pathway, Wnt pathway, mitogen-activated protein kinase (MAPK) pathway and Notch pathway [105]. Aberrant Notch pathway methylation has also been associated with BAV and cardiomyopathy [20].

**Table 1.** **Differentially methylated genes in common CHD subtypes**.

CHD subtype	Gene(s)	Methylation status
TOF	*NKX2-5*, *HAND1*, *EGFR*, *EVC2*, *TBX5*, *CFC1B*	Hypermethylated
	*NR2F2*	Hypomethylated
DORV	*ZIC3*, *NR2F2*	Hypermethylated
BAV	*NOTCH1*, *AXIN1*, *EGFR*, *ENG*, *GATA5*, *NKX2-5*, *NOS3*, *PDIA2*, *TGFBR2*	Hypermethylated
CoA	*TGFB1*, *SMAD1*	Hypermethylated
VSD	*TBX20*, *LY9*, *SLC9A3R2*	Hypermethylation
	*AXIN1*, *CBFA2T3*, *EPHA8*	Hypomethylation
AVSD	*CBFA2T3*, *EPHA8*	Hypermethylation
	*LY9*, *SLC9A3R2*	Hypomethylation

CHD, congenital heart disease; TOF, tetralogy of Fallot; DORV, double-outlet right ventricle; BAV, bicuspid aortic valve; CoA, coarctation of the aorta; VSD, ventricular septal defect; AVSD, atrioventricular septal defect; *HAND1*, heart and neural crest derivatives expressed 1; *EVC2*, EvC ciliary complex subunit 2; *TBX5*, T-box transcription factor 5; *CFC1B*, cripto, FRL-1, cryptic family 1B; *NR2F2*, nuclear receptor subfamily 2 group F member 2; *ZIC3*, Zic family member 3; *NOTCH1*, notch receptor 1; *ENG*, endoglin; *GATA5*, GATA binding protein 5; *NOS3*, nitric oxide synthase 3; *pDIA2*, protein disulfide isomerase family A member 2; *TGFBR2*, transforming growth factor beta receptor 2; *TGFB1*, transforming growth factor beta 1; *SMAD1*, SMAD family member 1; *TBX20*, T-box transcription factor 20; *LY9*, lymphocyte antigen 9; *SLC9A3R2*, SLC9A3 regulator 2; *CBFA2T3*, CBFA2/RUNX1 partner transcriptional co-repressor 3; *EPHA8*, EPH receptor A8.

In DORV, the promoter hypermethylation of Zic family member 3 (*ZIC3*) and nuclear receptor subfamily 2 group F member 2 (*NR2F2*) has been observed, correlating with reduced gene expression [106]. *ZIC3*, a zinc-finger transcription factor, regulates left–right body axis formation and cardiac development through inhibition of the Wnt pathway, which has also been implicated in TOF [105, 107]. *NR2F2*, a member of the steroid thyroid hormone superfamily of nuclear receptors essential for cardiac development [108], has been linked not only to DORV but also to AVSD and VSD. Interestingly, the *NR2F2* promoter was hypomethylated in TOF without significant RNA expression changes, suggesting context-dependent epigenetic regulation [104, 108].

BAV has also been studied extensively in the context of DNA methylation. Notch Receptor 1 (*NOTCH1*), a key regulator of valvulogenesis, is hypermethylated in BAV and may contribute to structural abnormalities and altered aortic wall architecture [109]. Genome-wide methylation analyses comparing BAV and tricuspid aortic valves identified 333 CpGs mapping to genes including *NOTCH1*, *AXIN1*, *EGFR*, endoglin (*ENG*), GATA binding protein 5 (*GATA5*), *NKX2-5*, nitric oxide synthase 3 (*NOS3*), protein disulfide isomerase family A member 2 (*PDIA2*), and transforming growth factor beta receptor 2 (*TGFBR2*), all of which exhibited significant differential methylation [110]. Similarly, a genome-wide analysis of blood samples from 24 newborns with nonsyndromic CoA and 16 controls identified 65 DMCs within 75 genes. These included transforming growth factor beta 1 (*TGFB1*) and SMAD family member 1 (*SMAD1*), both of which are crucial for cardiac development, as well as genes involved in glucocorticoid signaling, suggesting novel mechanisms of CoA pathogenesis [111].

## 5. DNA Methylation and Fetal Programming of Cardiovascular Diseases in Late Life

The concept of fetal programming suggests that the prenatal environment, particularly maternal health and exposures, can “program” the epigenome of the fetus, influencing both congenital malformations and later susceptibility to CVDs [112]. Among epigenetic mechanisms, DNA methylation is the most extensively studied [113]. By regulating gene expression, DNA methylation exerts profound influence on CVD and their risk factors in offspring, including hypertension, pulmonary vascular dysfunction, atherosclerosis, inflammation, and oxidative stress [114, 115, 116, 117]. Unlike fixed genetic mutations, epigenetic modifications are dynamic and responsive to nutritional, metabolic, and pharmacologic influences, making them promising targets for preventive strategies [118]. Maternal metabolic syndrome, which includes hypertension, diabetes mellitus, and dyslipidemia, represents a major prenatal condition capable of increasing adverse pregnancy outcomes and exerting substantial effects on fetal development [119] (Fig. 2). These maternal metabolic disturbances have lasting effects on the fetal epigenome, shaping cardiac development and predisposing offspring to long-term cardiovascular dysfunction, thereby linking fetal life with adult disease outcomes [120].

Pregnancy-induced hypertension provides a clear example of maternal influence on fetal methylation patterns. In umbilical cord blood from affected pregnancies, 560 DMRs have been identified, with 374 hypermethylated and 186 hypomethylated sites. These alterations mapped to nine genes implicated in cardiovascular development and disease, including v-abl Abelson murine leukaemia viral oncogene homologue 1 (*ABL1*), calcium voltage-gated channel auxiliary subunit alpha 2 delta 4 (*CACNA2D4*), guanylate cyclase 1 soluble subunit alpha 2 (*GUCY1A2*), laminin subunit alpha 2 (*LAMA2*), NADH dehydrogenase (ubiquinone) 1beta subcomplex 6 (*NDUFB6*), phospholipase C beta 1 (*PLCB1*), protein kinase cGMP-dependent 1 (*PRKG1*), TANK-binding kinase 1 (*TBK1*) and vav guanine nucleotide exchange factor 3 (*VAV3*) [121]. These findings suggest that maternal hypertensive disorders leave persistent epigenetic marks that may predispose offspring to cardiovascular dysfunction in later life.

In addition to pregnancy induced hypertension, gestational diabetes mellitus (GDM) has emerged as a potent modifier of the fetal cardiac epigenome. Our previous work demonstrated that GDM exposure induces an ischemia-sensitive cardiac phenotype in offspring through alterations in DNA methylation [117]. Placental tissue from GDM pregnancies revealed 8657 differentially methylated CpGs compared with controls, with enrichment of pathways related to CVD. Ingenuity pathway analysis identified 91 genes linked to CVD risk [122, 123]. In umbilical cord blood, genes such as ATP synthase F1 subunit alpha (*ATP5A1*), microfibril associated protein 4 (*MFAP4*), and solute carrier family 17 member 4 (*SLC17A4*), which are associated with oxidative stress defense and cardiovascular complications, were differentially methylated in GDM-exposed offspring [124]. These findings support the hypothesis that maternal hyperglycemia reprograms fetal gene expression through methylation changes, thereby increasing susceptibility to CVD in adulthood.

Maternal dyslipidemia also contributes to fetal cardiovascular risk via DNA methylation. In offspring exposed to maternal hypercholesterolemia, the CpG island within the sterol regulatory element binding transcription factor 2 (*SREBF2*) gene was found to be significantly hypermethylated in fetal aortic tissue [125]. *SREBF2* encodes a transcription factor that regulates cholesterol, lipid, and glucose metabolism [126]. Dysregulation of this pathway has been implicated in a wide range of cardiometabolic disorders, including hypertension, atherosclerosis, dyslipidemia, obesity, insulin resistance, and type 2 diabetes [127, 128]. Thus, altered *SREBF2* methylation provides a mechanistic link between maternal cholesterol levels and long-term cardiovascular dysfunction in offspring.

Other maternal exposures also affect fetal DNA methylation in ways that predispose to CVD. For example, maternal electronic cigarette exposure has been shown to worsen pulmonary hypertension in offspring through hypomethylation of key regulatory genes [61]. These findings underscore that maternal exposures beyond traditional risk factors can exert lasting cardiovascular effects through epigenetic mechanisms.

Collectively, these studies illustrate that DNA methylation serves as a critical mediator of fetal programming. Maternal conditions such as hypertension, diabetes, and hyperlipidemia leave stable epigenetic marks that shape offspring cardiovascular physiology, thereby predisposing to disease later in life. Recognition of these pathways highlights the potential of maternal risk factor modification and targeted epigenetic interventions as strategies to reduce the burden of CVD across generations.

## 6. Mapping Congenital Heart Diseases-to-Adult Cardiovascular Dysfunction Trajectories With Single-Cell Epigenomics

Single-cell epigenomic technologies have fundamentally advanced the understanding of cardiac developmental biology by enabling high-resolution mapping of DNA methylation dynamics across discrete cellular lineages. These approaches uncover regulatory mechanisms that are obscured in bulk analyses and illuminate how epigenetic heterogeneity orchestrates cardiogenesis.

In a recent study, a genomic DNA methylation reporter system was employed to visualize real-time methylation changes during induced pluripotent stem cells (iPSC) cardiac differentiation [129]. By fusing CpG regions of genes such as SRY-Box transcription factor 2 (*Sox2*) and Cyclin dependent kinase 1 (*Cdk1*) with the methylation-sensitive Snrpn minimal promoter, the authors tracked reporter fluorescence as a proxy for methylation status. This live-cell imaging approach revealed that promoter hypermethylation of *Sox2* and *Cdk1* correlated with decreased expression during cardiomyocyte differentiation, highlighting how DNA methylation dynamically regulates both stemness and cell cycle exit in developing cardiac cells. Complementing such reporter-based methods, single-cell multi-omics platforms like snm3C-seq allow simultaneous profiling of DNA methylation and 3D chromatin architecture within the same cell. In a landmark study by Chen and colleagues [130], snm3C-seq was applied to human subcutaneous adipose tissue, uncovering cell-type-specific methylation patterns and chromatin compartmentalization. Although focused on adipose biology, the methodology is directly applicable to cardiac tissue, where it could reveal how methylation dynamics coordinate with spatial genome organization to drive lineage-specific gene expression during heart development. Moreover, single-cell RNA sequencing has been integrated with epigenomic analyses to link methylation states to transcriptional outputs. In the heart failure study [131], scRNA-seq of non-cardiomyocyte heart cells identified fibroblast-endothelial crosstalk mediated by *ANGPTL4*, a gene likely under epigenetic control. Such integrative analyses highlight how methylation changes in specific cell types—such as fibroblasts or endothelial cells—can influence cardiac pathophysiology through altered gene expression and cell communication.

Collectively, cardiac development is guided by tightly orchestrated, lineage-specific epigenetic reprogramming. Hypermethylation of proliferation-related genes drives cardiomyocyte cell-cycle exit, while hypomethylation of structural and metabolic genes promotes maturation. Single-cell methylation maps identify lineage-defining regulatory regions implicated in CHD and later cardiovascular risk. Applying single-cell epigenomic approaches to CHD may reveal early methylation defects underlying structural abnormalities and phenotypic variability. Longitudinal profiling across developmental stages could clarify how fetal epigenetic disturbances predispose to maladaptive remodeling, arrhythmias, or heart failure. Integrating methylation, chromatin architecture, and transcriptional data may establish a continuous epigenetic trajectory linking CHD with adult cardiovascular dysfunction and support precision-based approaches aimed at modifying pathogenic epigenetic states.

## 7. Conclusions

DNA methylation plays a crucial role in regulating cardiac development and the fetal programming of CVDs. This review emphasizes the significant influence of maternal factors on the offspring’s epigenome, specifically how DNA methylation reprograms genes involved in cardiogenesis, thereby contributing to CHDs and the predisposition to adult cardiovascular dysfunction.

Future research should focus on understanding the specific ways in which maternal environmental exposures, such as smoking, diabetes, and hypertension, lead to DNA methylation changes at critical cardiac loci during pregnancy, and how these changes influence the development of CHDs. Investigating the mechanisms by which early epigenetic alterations, such as methylation of proliferation-related and structural genes, contribute to later-life cardiovascular conditions like arrhythmias, heart failure, and vascular dysfunction is another critical area for exploration.

Moreover, the potential of maternal lifestyle modifications or pharmacological interventions to reverse or mitigate these DNA methylation changes warrants investigation. It is crucial to determine whether such interventions can reduce the risk of CHDs and improve cardiovascular health outcomes in offspring.

In addition, applying single-cell epigenomic technologies, in combination with chromatin profiling, could help identify early biomarkers for cardiovascular dysfunction in individuals with a history of adverse fetal programming. These approaches may provide valuable insights into the epigenetic trajectory linking CHDs with adult CVDs.

By addressing these questions, the field can advance towards effective prevention and therapeutic strategies to improve cardiovascular health across the lifespan.
